# Editorial: Learning in Social Context: The Nature and Profit of Living in Groups for Development

**DOI:** 10.3389/fpsyg.2017.00336

**Published:** 2017-03-09

**Authors:** Ildikó Király, David Buttelmann

**Affiliations:** ^1^Cognitive Psychology Department, Social Minds Research Group, Eötvös Loránd UniversityBudapest, Hungary; ^2^Department of Developmental Psychology, University of BernBern, Switzerland

**Keywords:** social learning, social categorization, cognitive development, understanding social relations, group processes

One of humans' most distinctive feature is their unique sociality. Research has shown that people are ready to use a variety of cues to draw distinctions between “us” and “them” (Over and Carpenter, 2012). Theories of social categorization share common assumptions: in-group bias may benefit an individual as it helps them to boost their own self-esteem (Tajfel and Turner, 1986) or provides an ideological ground for oppressing others (Sidanius and Pratto, 1993).

Past research in developmental psychology has already provided insight into children's representations of the social world. It has been shown that infants as young as only a few months of age categorize others based on gender (Quinn et al., 2002). They even do so for language (Kinzler et al., 2007), which has been identified as a reliable indicator of group-membership for infants. While there is emergent evidence that already infants form “social categories,” little is known about the fact whether infants' social categories reflect an “in-group” preference *per se*, or a preference for people sharing traits with those in their environment.

The central question of this research topic focused on the role of the ability to categorize social partners in the environment for the developing mind. More precisely, we wanted to see whether this ability influences epistemic development as well, beyond the enrichment of social-emotional competencies.

Relatedly, the first question the research topic covers is how children understand the relevance and source of group cohesion. In history, kinship relations have prominently marked the formation of social groups. Yet, experiments have not examined children's knowledge of and reasoning about kinship. The findings of Spokes and Spelke suggest that an explicit understanding of kinship develops slowly over the preschool years. They show that children handle kinship very similarly to how they handle other close social relations, like friendship, from early on. Another cue to group formation might be an individual's allocation of resources to others. More specifically, fairness preference is one important phenomenon with respect to differences in behavior dependent on social relations. The study of Li et al. provides further evidence on the early preference of fair distribution among social partners, and its dependence on disadvantageous positions of the self. Finally, another important cue to group cohesion might be behavioral consensus. Zhao et al. reveal an increase in sensitivity to behavioral consensus in 2- to 5-year-old children and their ability to use this as a marker of group membership. However, in contrast to most previous studies, these authors highlight contexts in which children seem to prefer to learn from unconventional individuals.

The aim of the review of Esseily et al. is to find out (1) how children orient preferences and actions toward social partners and (2) how these preferences change over early ontogeny. They highlight the role language plays in guiding categorization relative to other cues such as age, race and gender. The authors explain this by the reliability of language regarding informing individuals about the speaker's group membership and consequently, her reliability as a source of culturally relevant information.

Following this idea empirically, Marno et al. show that 12- and already 5-month-old infants selectively attend to informants who are native speakers of their language. The authors suggest that by this young, children can maximize the possibility to acquire potentially important cultural knowledge. van Schaik et al. investigate the effect of novel group membership on young children's motor behavior during a simultaneous movement-observation and -execution task. Their research focus is on online motor copying, in order to understand the influence of group membership on basic coordination processes. Their results reflect an effect of heightened attention toward interaction with an out-group member. This provides important evidence that novel group membership—even if induced by arbitrary or minimal cues—dynamically influences interactive behavior. The findings of these two studies together give new insight into the impact of an opponent's language group membership on children's basic cognitive processes.

Investigating more complex action planning and execution, Krieger et al. ask whether difference in group membership between two models would trigger variation in children's imitative tendencies. They provide empirical evidence on that difference in the model's physical appearance (i.e., race) is not sufficient to elicit an in-group-out-group effect in terms of preference to follow one of the demonstrators behaviorally. In a similar vein, the purpose of Oláh et al.'s study is to investigate more enhanced processes that are cultural in their nature. The authors focused on tool use and show that tool function learning is dependent on demonstrator's group membership, in other words, function learning occurs more prominently when it is introduced as part of a cultural knowledge context.

The above studies provide insight into the characteristics of human-specific learning processes in addition to socio-emotional motivation aspects by showing that children are sensitive to a social partner's group membership. Dependent on task requirements, children flexibly exploit the advantage group membership could provide, like in case of learning from more knowledgeable partners, or paying more attention to potential outgroup members, while ignoring group membership if it delivers no benefit with respect to development.

The last study in this special issue goes beyond the investigation of the possible consequences of the detection of group membership on children's preferences and learning and shows how these consequences can be changed. Tunçgenç and Cohen focus on the robustness of the in-group bias. Their participants—minimally divided into groups—performed movements either synchronously or non-synchronously to an in-group or an out-group member. Self-report and behavioral measures point toward a bonding effect for synchronous movement and, consequently, a decrease in in-group bias.

In sum, this research topic contributes to the understanding of the epistemic function of social category formation by showing that: (1) children use specific cues, like kinship, fairness and consensus to understand group cohesion; (2) once they figured out who is in-group and who is out-group, they attend, act and learn selectively; yet (3) these consequences can be changed by induced synchronous behavior.

## Author contributions

All authors listed, have made substantial, direct and intellectual contribution to the work, and approved it for publication.

## Funding

This work received financial support from OTKA (grant 109 352, PI: IK).

### Conflict of interest statement

The authors declare that the research was conducted in the absence of any commercial or financial relationships that could be construed as a potential conflict of interest.
